# Editorial Comment to Laparoscopic transmesenteric pyeloplasty and isthmusectomy for adult horseshoe kidney with recurrent symptomatic hydronephrosis

**DOI:** 10.1002/iju5.12201

**Published:** 2020-07-21

**Authors:** Francesco Chiancone

**Affiliations:** ^1^ Urology Department Cardarelli Hospital Naples Italy

Horseshoe kidneys are found in about 0.25% of the population. They are commonly associated with other abnormalities like ureteropelvic junction obstruction (UPJO) and vescicoureteral reflux. UPJO can be caused by anomalous renal blood supply, high‐inserting ureter into the renal pelvis or its abnormal course over the isthmus, and intrinsic factors.

Lu *et al*. described the mini‐invasive surgical treatment of UPJO in five patients with horseshoe kidneys.^1^ Isthmusectomy and Anderson‐Hynes dismembered pyeloplasty were performed laparoscopically with a trans‐mesenteric approach in all cases without bowel complications. All procedures were successful and patients were discharged within 5.7 days. Despite lack of post‐operative functional data, ultrasonography or urography showed good outcomes.

Standard techniques suggest isthmusectomy in order to obtain a more physiological urine drainage. Despite this, Shadpour *et al*.^2^ performed isthmusectomy only in 1 out of 15 (7%) horseshoe kidneys with a long‐term success rate of 93.3%.

There have been continuous debate in literature about the use of a retroperitoneal or transperitoneal approaches for the management of UPJO. A recent meta‐analysis concluded that both approaches are associated with high success and low complication rates, but transperitoneal pyeloplasty provides a shorter procedure time and lower conversion rate.^3^ In horseshoe kidneys transperitoneal approach seems to be preferred^2^ even if Wang *et al*.^4^ described a 100% clinical success rate in a series of retroperitoneal dismembered pyeloplasty associated to the division of the isthmus.

The robotic approach has been documented in only seven cases in the literature.^2^ Three adult patients were treated with dismembered pyeloplasty without isthmusectomy. Two pediatrics patients were treated with dismembered pyeloplasty and the other two with “vascular hitch” (Hellstrom) procedure.^5^ No data are available concerning isthmusectomy in this series of pediatrics patients. The higher costs related to robotic procedures probably did not allow the widespread use of this technique.

Multicentric randomized controlled trials with a longer follow‐up period are needed to establish an optimal treatment technique for this rare disorder.

## Conflict of interest

The author declares no conflict of interest.
